# Involvement of Synaptic Genes in the Pathogenesis of Autism Spectrum Disorders: The Case of Synapsins

**DOI:** 10.3389/fped.2014.00094

**Published:** 2014-09-04

**Authors:** Silvia Giovedí, Anna Corradi, Anna Fassio, Fabio Benfenati

**Affiliations:** ^1^Department of Experimental Medicine, University of Genova, Genova, Italy; ^2^Department of Neuroscience and Brain Technologies, Fondazione Istituto Italiano di Tecnologia, Genova, Italy

**Keywords:** autism, synaptopathies, synaptic vesicles, synaptic transmission, social behavior, human mutations, knockout mice

## Abstract

Autism spectrum disorders (ASDs) are heterogeneous neurodevelopmental disorders characterized by deficits in social interaction and social communication, restricted interests, and repetitive behaviors. Many synaptic protein genes are linked to the pathogenesis of ASDs, making them prototypical synaptopathies. An array of mutations in the synapsin (Syn) genes in humans has been recently associated with ASD and epilepsy, diseases that display a frequent comorbidity. Syns are pre-synaptic proteins regulating synaptic vesicle traffic, neurotransmitter release, and short-term synaptic plasticity. In doing so, Syn isoforms control the tone of activity of neural circuits and the balance between excitation and inhibition. As ASD pathogenesis is believed to result from dysfunctions in the balance between excitatory and inhibitory transmissions in neocortical areas, Syns are novel ASD candidate genes. Accordingly, deletion of single Syn genes in mice, in addition to epilepsy, causes core symptoms of ASD by affecting social behavior, social communication, and repetitive behaviors. Thus, Syn knockout mice represent a good experimental model to define synaptic alterations involved in the pathogenesis of ASD and epilepsy.

Autism spectrum disorders (ASDs) represent a wide array of neurodevelopmental disorders characterized by restricted interest, defective social interactions, repetitive behaviors, and deficit in language and verbal communication that manifest within the first 3 years of life (1, 2). The complexity of ASD is evident both at the levels of symptoms variability and of causative factors. An important genetic contribution has been observed for ASD, however, the mechanism of inheritance remains largely unknown (1). More than 500 genes have been associated with different forms of autism, but each of them account only for the minority of ASD cases and indeed environmental contributions and other modulating factors, as environment-genetic interplay and epigenetic modifications, are emerging as potential risk factors for ASDs (3).

## Involvement of Synaptic Proteins in the Pathogenesis of ASDs

Autism spectrum disorders may result from mutations in a large array of genes having roles in various physiological processes such as chromatin remodeling, translation, metabolism, and synaptic functions. In experimental models of ASD, a common breakdown appears to occur at the level of synapse formation and stabilization, as well as of the ability of synapses to be modified by experience through plasticity mechanisms. Synapse dysfunctions are also at the convergence between ASD and other neuropsychiatric disorders with unknown etiology, such as schizophrenia and intellectual disabilities (4). In addition, ASDs frequently occur together with epilepsy and there may be common underlying mechanisms as well as common genetic and environmental risk factors. Synapse maturation and function rely on a vast array of compartmentalized protein–protein interactions that allow fidelity in neurotransmitter release and synaptic vesicle (SV) cycling at the pre-synaptic site and in neurotransmitter receptor localization and signaling at the post-synaptic site. Moreover, synaptic adhesion molecules link and stabilize pre- and post-synaptic sites and control synaptic modification induced by plasticity.

**Figure 1:**
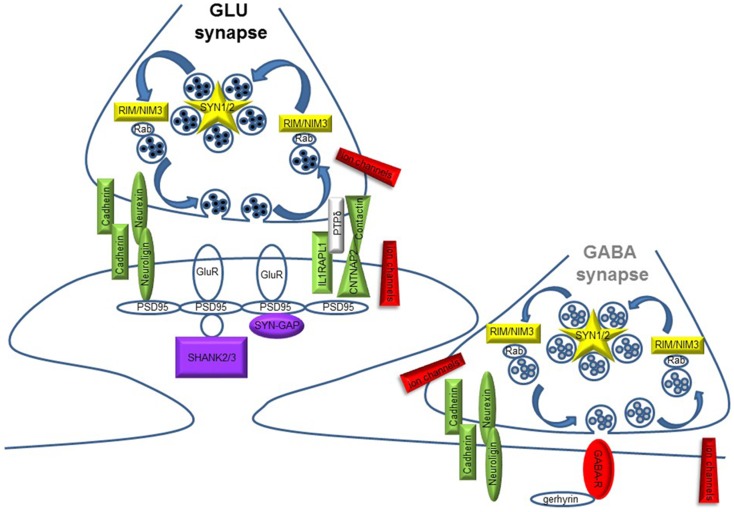
**Schematic diagram illustrating pre- and post-synaptic gene products implicated in ASD**. Glutamate (GLU) and GABA synapses are shown. Different colors code for synaptic function: yellow, synaptic vesicle cycling; green, synapse formation and maintenance; red, neuronal excitability and neurotransmission; violet, glutamate receptors (GluR) signaling/trafficking.

The synaptic theory for autism originally stems from the identification of ASD mutations in the Neuroligin genes [NLGN3 and NLGN4X; (5, 6)] coding for synaptic adhesion molecules expressed at the post-synaptic site. Since then, a growing repertoire of synaptic genes, coding for both pre- and post-synaptic proteins, have been implicated in non-syndromic ASDs: synaptic adhesion molecules [neuroligins, neurexins, cadherins, contactins and contactin-associated protein-like 2, or CNTNAP2; (7–9)], synaptic scaffold proteins [PROSAP/SHANK gene family; (10)], ion channels, and neurotransmitter receptors (11–13). Moreover, mutations in additional synaptic genes, such as the pre-synaptic RIMS3/NIM3 and the post-synaptic IL1RAPL1 and SynGAP1, involved in either SV organization or synapse formation, have been recently associated with ASD cases [(14–17); Figure 1 and Table 1]. The identification of synaptic genes implicated in ASDs is expanding together with the characterization of the respective animal models bearing mutations or deletions in these genes. These models allow a more systematic analysis to study the role of those genes in ASD etiology, to discover the biological mechanisms underlying autistic behaviors and evaluate the efficacy of new potential treatments (18–21).

**Table 1:** **Synaptic genes associated with ASD**.

Gene	Name	Chromosomal locus	Phenotype	Function
SYN1	Synapsin1	Xp11.23	ASD, epilepsy	Synaptic vesicle cycling
SYN2	Synapsin2	3p25	ASD, epilepsy	Synaptic vesicle cycling
RIMS3	Regulating synaptic membrane exocytosis 3	1p34.2	ASD	Synaptic vesicle cycling
CACNA1E	Calcium channel, voltage-dependent R type, alpha 1E subunit	1q25.3	ASD	Neurotransmission
CACNB2	Calcium channel, accessory beta2 subunit	10p12	ASD	Neurotransmission
SCN1A	Voltage-regulated sodium channel type 1	2q24.3	Dravet syndrome, ASD	Neuronal excitability
SCN2A	Voltage-regulated sodium channel type 2	2q24.3	ASD, epilepsy	Neuronal excitability
SCN3A	Voltage-regulated sodium channel type 3	2q24	ASD, epilepsy	Neuronal excitability
KCNMA1	Potassium calcium-activated channel, subfamily M, alpha member 1	10q22.3	ASD	Neuronal excitability
KCNMB4	Potassium calcium-activated channel, subfamily M, beta member 4	12q	ASD	Neuronal excitability
KCNQ3	Potassium voltage-gated channel	8q24	ASD, epilepsy	Neuronal excitability
KCNQ5	Potassium voltage-gated channel	6q14	ASD, epilepsy	Neuronal excitability
KCND2	Potassium voltage-gated channel	7q31	ASD, epilepsy	Neuronal excitability
NRXN1	Neurexin1	2p16.3	ASD, schizophrenia	Cell-adhesion
NLGN3	Neuroligin3	Xq13.1	ASD	Cell-adhesion
NLGN4X	Neuroligin4	Xp22.32–p22.31	ASD, intellectual disability	Cell-adhesion
CNTNAP2	Contactin-associated protein-like 2	7q35	ASD, intellectual disability, epilepsy schizophrenia	Cell-adhesion
CDH5	Cadherin 5	16q22.1	ASD	Cell-adhesion
CDH8	Cadherin 8	16q22.1	ASD	Cell-adhesion
CDH9	Cadherin 9	5p14	ASD	Cell-adhesion
CDH10	Cadherin 10	5p14.2	ASD	Cell-adhesion
CDH11	Cadherin 11	16q21	ASD	Cell-adhesion
CDH13	Cadherin 13	16q23.3	ASD	Cell-adhesion
CDH15	Cadherin 15	16q24.3	ASD, intellectual disability	Cell-adhesion
PCDHB4	Protocadherin beta4	5q31	ASD	Cell-adhesion
PCDH10	Protocadherin delta 10	4q28.3	ASD	Cell-adhesion
PCDH19	Protocadherin delta 19	Xq22.1	ASD, intellectual disability	Cell-adhesion
CNTN4	Contactin 4	3p26.3	ASD, intellectual disability	Cell-adhesion
CNTN5	Contactin 5	11q22.1	ASD	Cell-adhesion
CNTN6	Contactin 6	3p26–p25	ASD	Cell-adhesion
IL1RAPL1	Interleukin 1 receptor accessory protein-like 1	Xp22.121.3	ASD, intellectual disability	Cell-adhesion
SHANK1	SH3 and multiple ankyrin domain protein 1	19q13.3	ASD	Glutamate receptor signaling
SHANK2	SH3 and multiple ankyrin domain protein 2	11q13.3	ASD	Glutamate receptor signaling
SHANK3	SH3 and multiple ankyrin domain protein 3	22q13.3	ASD	Glutamate receptor signaling
SYNGAP1	Synaptic Ras GTPase activating protein 1	6p21.3	ASD	Glutamate receptor signaling
GABRG3	Gamma3 subunit of GABA-A receptor	15q12	ASD	Neurotransmission

## ASD is Associated with Dysfunctions in Cortical Circuits That also Predispose to Epilepsy

Autism spectrum disorder-like phenotypes are observed in a wide variety of neurological and neurodevelopmental disorders, including epilepsy, Rett syndrome, Fragile X Syndrome, Tuberous Sclerosis, or Fetal Anticonvulsant Syndrome, which are characterized by an imbalance of the excitatory/inhibitory tone. This aspect is consistent with the very high prevalence of epilepsy in autistic patients (about 25%) with respect to the average prevalence of 1% in the general population. Characteristic ASD phenotypes are associated with impairments or gains of GABAergic transmission (22–25).

Decreases in the GABA synthesizing enzyme GAD and a reduction in quantal size have been reported in the experimental model of Rett syndrome, the Mecp2 knockout (KO) mice (26). Various impairments in GABAergic function, including deficient GABAergic circuitry, decreased expression of GABA_A_ receptor subunits (particularly α5 and γ subunits) and of the tonic GABA current, have been observed in mice lacking FMRP, a recognized experimental model of Fragile X Syndrome (27). The chromosomal region 15q11–q13, which is deleted or duplicated in 1–2% of idiopatic ASD patients, contains a cluster of genes encoding GABA receptor subunits [Gabra5, Gabrb3, and Gabrg3; (11)] and deletion of the Gabrb3 gene in mice, encoding for the GABA_A_ β3 receptor subunit, leads to an ASD-like phenotype (28). Moreover, Reeler mice, lacking the protein reelin that is expressed in cortical interneurons, display an ASD phenotype that is associated with a decrease in GABA turnover (29, 30). Mice lacking synapsins (Syns) also display a primary impairment in GABA release dynamics that is associated with an ASD-like phenotype [see below; (31–35)]. Taken together, all these experimental data indicate that GABA systems are major actors in the development and functioning of cortical networks, and that their dysfunction can lead to altered development and/or function of cortical circuits resulting in epilepsy, ASD, or both. Moreover, the role of GABAergic dysfunctions in ASD pathogenesis is complex given the switch between excitatory and inhibitory GABA transmission that occurs during development and the multiple sites of action of GABA in mature neuronal networks, where it acts on predominantly post-synaptic GABA_A_ receptors, extrasynaptic GABA_A_ receptors regulating excitability and predominantly pre-synaptic GABA_B_ receptors modulating glutamate release and short-term plasticity properties of excitatory synapses. Thus, disruption or dysfunction of GABAergic systems may delay critical periods in specific brain regions and perturb γ-oscillations implicated in high cognitive functions. Given the importance of the excitation/inhibition balance in the activity-dependent formation and plasticity of neocortical networks, we here review on the role of the Syns, a family of pre-synaptic proteins regulating release and plasticity in inhibitory and excitatory synapses, in the etiology of ASDs and the use of Syn KO animals as a model for these complex neuropsychiatric disorders.

## Synapsins

The Syns are a family of abundant neuronal phosphoproteins that participate as regulators in synaptic transmission and plasticity, as well as in neuronal development [see Ref. (36, 37), for review]. The family is composed of 10 homologous proteins: Syn Ia–b, Syn IIa–b, and Syn IIIa–f (38–40), encoded in mammals by alternative splicing of three distinct genes (*SYN1, SYN2*, and *SYN3*) mapping on distinct chromosomes (chromosome X, 3, and 22, respectively) in human and mouse. Notably, Syn III is the most precociously expressed isoform that has a role in the early phases of neural development and is downregulated in mature neurons (40). On the other hand, Syn I and Syn II are expressed at low levels at birth and their expression progressively increases along synaptogenesis to reach a stable plateau at 1–2 months of life, approximately the time window epilepsy appears (41).

All Syn isoforms display a domain structure with the NH_2_-terminal region, highly conserved across isoforms and species, divided in domains A, B, and C, and the COOH-terminal portion, more divergent, composed of different spliced domains [D–I; (38)]. Many of these isoforms share consensus sequences for phosphorylation by several protein kinases, which all contribute to the modulation of Syn function (36). Domain A contains the phosphorylation site for PKA and CaMKI/IV that modulates the reversible association of Syn with SVs. Domain B, less conserved and considered as a link region, contains phosphorylation sites for MAPK/Erk, which also causes the redistribution of Syn from SVs to the cytosol. Domain C, a large central region of about 300 amino acids, mediates the interaction with actin filaments and SVs and promotes SV clustering by inducing Syn homo/hetero-dimerization (42, 43). This domain is phosphorylated by the tyrosine kinase Src (44) and contains residues mediating the binding to ATP (45). The sequences of Syn isoforms at the COOH-terminal region diverge (domain D in Syn Ia and Ib, domain G in Syn IIa and IIb, domain H in Syn IIa, and domain J in Syn IIIa), although they all bear proline-rich regions binding to several SH3-containing proteins (46, 47), and additional phosphorylation sites for CaMKII, MAPK/Erk, and cdk1/5, which affect the biochemical properties of Syn I resulting in a drastic reduction of its binding to both actin and SVs (48, 49). Finally domain E, highly conserved and common to all “*a*” isoforms, modulates Syn targeting to the pre-synaptic terminals and SV trafficking (50–53).

The best-characterized function of Syns is to control SV trafficking and modulate neurotransmitter release at the pre-synaptic terminal. The fine regulation of the balance between the reserve and the readily releasable pool of SVs is strictly controlled by Syn site-specific phosphorylation in response to stimulation, which modulates Syn association with SVs, actin cytoskeleton, and other synaptic proteins, and leaves SVs free to move close to the active zone and undergo fusion. Besides the function of Syns in these pre-docking stages of neurotransmission, recent data, supported by the fact that at least part of Syn do not dissociate from SVs upon fusion, strongly indicate that Syns play a role in the final post-docking stages of exocytosis, including SV priming, fusion, and recycling of the synaptic membrane in the area surrounding the active zone [see Ref. (36), for review].

Beyond the role in synaptic transmission, the various Syn isoforms play an important role in neuronal growth and synaptogenesis. Lack of Syn I or Syn II was shown to impair neurite outgrowth during the first days *in vitro* (54), while downregulation or ablation of Syn III caused an impairment in the development of axons at early stages in culture (55). Moreover, clear-cut structural and physiological defects were observed in the pre-synaptic terminals of Syn KO neurons (56–58), confirming the role of Syn isoforms in the modulation of synapse formation, maintenance, and rearrangement [see Ref. (37), for review].

## Synapsin KO Mice are Epileptic

Knockout mice for either Syn gene are viable and fertile, have a normal life expectancy and brains of normal size and gross structure. As Syns are involved in the regulation of the excitability of neuronal networks, it is not surprising that the impairment of Syn function can result in epilepsy. Syn I KO, Syn II KO, Syn I/II double KO, and Syn I/II/III triple KO are all prone to epileptic seizures that appear approximately at 2–3 months of age, and progressively aggravate with aging and the number of Syn genes ablated [(59, 60); see Ref. (61) for review]. The fact that epilepsy does not appear at birth after ablation of Syn I or Syn II genes and that Syn III KO mice are not epileptic can be explained by the specific expression profile of the three Syn genes during development (55, 62). It is therefore likely that mature synapses require physiological levels of both Syn I and Syn II to achieve a stable excitation/inhibition balance during activity, while Syn III seems to be dispensable in this respect. In general, the loss of Syns disrupts the reserve pool of SV and alters release dynamics. However, the pre- and post-docking effects of the Syns differentially affect excitatory and inhibitory neurons. This, together with the selective distribution of the various Syn isoforms in distinct neuronal populations and the non-overlapping functions of Syn isoforms on neurotransmitter release, can result in an imbalance between excitatory and inhibitory synaptic transmission, both under conditions of basal activity and of high-frequency stimulation, potentially leading to epileptogenesis (31, 33, 34).

## Synapsin KO Mice Display an ASD-Like Phenotype

Although it is not an easy task to translate the complex symptoms of human ASD into mouse behaviors, the study of the phenotype of mice bearing deletions in the genes found to be mutated in ASD patients is fundamental for the understanding of how dysfunction of single components of the synaptic protein network may result in a general functional impairment that generates the disease. Such mouse models of ASDs should display decreased interest toward the environment, impaired sociability, and social interactions/communication, as well as repetitive behaviors.

Synapsin KO mice have generally preserved cognitive functions. A prospective study performed on Syn I and Syn II KO mice revealed that cognitive and emotional performances are not altered before the onset of epilepsy both in terms of spatial memory, object recognition, and emotional memory. Only later on, during aging and in the presence of an overt epileptic phenotype, behavioral deficits in emotional memory in both genotypes and spatial memory in Syn II KO mice emerged with respect to wild type controls, and were associated with neuronal loss and gliosis in the cortex and hippocampus (63). On the other hand, Syn III KO mice that are not epileptic, exhibit only minor alterations in spatial memory, object recognition, fear conditioning, and fear-potentiated startle (40, 64).

When an array of socially directed behaviors (social interaction and novelty, social recognition and social dominance, social transmission of food preference, and social memory) were investigated in Syn I, Syn II, and Syn III KO mice before (2-months old) and after (6-months old) the appearance of epilepsy (in Syn I and Syn II KO mice), it was immediately clear that mice presented various impairments in social behaviors and repetitive behaviors well before the appearance of the epileptic phenotype (65).

**Figure 2:**
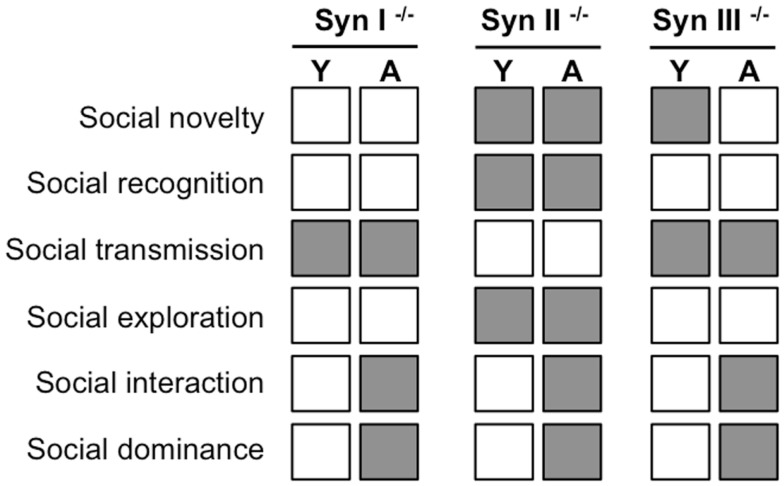
**Summary of the abnormalities in social behavior (gray squares) observed in young (Y, 2-month old) and adult (A, 6-month old) Syn I, Syn II, and Syn III KO mice with respect to the behavior of age-matched controls sharing the same genetic background [data from Ref. (65)]**. Adults, but not young, Syn I and Syn II KO mice are epileptic.

Synapsin III mice had the mildest phenotype and showed impairments only in social interactions with an intruder, a decreased social dominance and a decreased social transmission of food preference. Syn I KO mice had an intermediate phenotype and had deficits in social and environmental exploration, social transmission, and an increased social dominance. Finally, Syn II KO had the more severe behavioral phenotype and exhibited significant deficits in virtually all social behaviors tested (with the exception of the social transmission of food preference) together with an increased social dominance and repetitive self-grooming behaviors (Figure 2). These data indicate that Syn KO mice represent an interesting animal model for ASDs. The physio-pathological importance of this model for the understanding of ASD pathogenesis is underlined by the occurrence of ASD-linked loss-of-function mutations in human SYN genes, as described in the following section.

## Mutations in the SYN Genes are Associated with ASD in Humans

**Figure 3:**
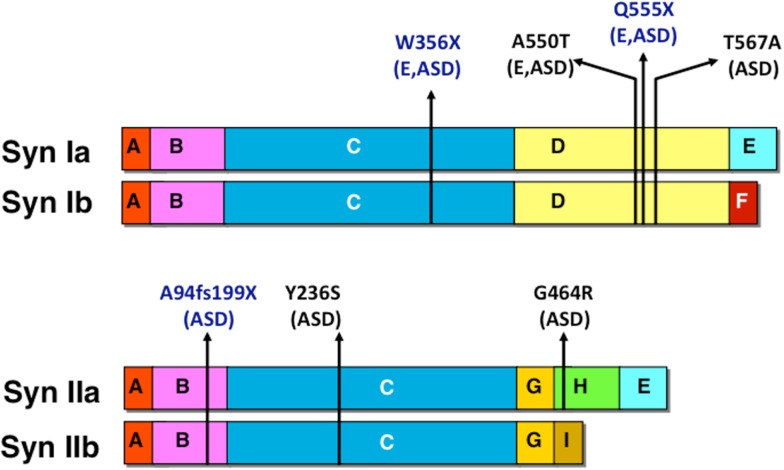
**Human mutations in SYN1 and SYN2 associated with epilepsy and/or ASD**. Nonsense and missense mutations are indicated in blue and black, respectively together with the associated pathology (E, epilepsy; ASD, autism spectrum disorder).

Autism spectrum disorder-associated mutations were found in both human SYN1 and SYN2 genes. Mutations in SYN1 were mainly identified in patients affected by both epilepsy and autism, whereas, mutations in SYN2 were observed in cases of ASD without association with epilepsy. The identified mutations are schematized in Figure 3. Two SYN1 nonsense mutations, causing truncations at protein level (W356X and Q555X), were identified in two large families with epilepsy with recessive X-linked transmission. In addition to epilepsy, few males carrying the mutations in SYNI also presented learning difficulties, low average IQ and three of them meet criteria for ASD (66, 67). The SYN1 missense mutation A550T was isolated in four patients: two with epilepsy, one with autism, and one with both, whereas, the missense mutation T567A was isolated in two individuals with ASD only. A frameshift (A94fs199X) and two missense (Y236S and G464R) mutations were identified in the SYN2 gene in three males affected by ASD (68). The mutation was transmitted by the non-affected mother. Although this phenomenon was observed only in a limited number of individuals, it is consistent with a recent report on the autosomal SHANK1 gene deletions associated with ASD in males but not females (69). Autosomal sex-limited expression, in addition to the mutation in X-linked genes, may contribute to the increased prevalence of ASD in males with respect to females. The exact mechanism at the basis of the higher penetrance in males remains to be determined.

## Effects of Human SYN Mutants Expressed in SYN KO Neurons

To get insight into the molecular mechanisms of the pathogenesis of these diseases, the physiological effects of the SYN genetic variants associated with ASDs or epilepsy (or both) were analyzed *in vitro* by expressing the mutants in primary hippocampal neurons from Syn I KO or Syn II KO mice and their effects on neuronal development, nerve terminal targeting, dynamics of exo-endocytosis, and synaptic transmission were studied and compared with wild type Syn.

For the W356X mutation in Syn I, the presence of a premature stop codon in the human SYNI transcript leads to nonsense-mediated mRNA decay (NMD). The few transcripts escaping NMD process give rise to a mislocalized and non-functional protein (70). On the contrary, the second nonsense mutation (Q555X) in Syn I does not lead to NMD and a truncated form of Syn I is expressed that lacks about a half of the D domain and the COOH-terminal domains E/F. The lack of D domain impairs its binding to SVs, its phosphorylation by CaMKII and MAPK/Erk and its interactions with SH3 domain-containing proteins such as PI3K, Src, endophilin, and intersectin (67). Neurons expressing the Q555X-Syn I showed a transient impairment of axonal outgrowth, but normal dendritic arborization, nerve terminal targeting, and synaptic density (67). However, the exocytosis was impaired and the size of the readily releasable and recycling pools of SVs decreased. Electrophysiological recordings from neurons expressing Q555X-Syn I mutant in Syn I KO background showed that, while the basal excitatory and inhibitory transmissions were equally depressed by the mutant, a clear imbalance in short-term plasticity was present with excitatory synapses showing markedly increased paired-pulse facilitation and post-tetanic potentiation as well as faster recovery from depression, whereas, inhibitory synapses displaying an enhanced post-tetanic depression and synaptic depression during sustained high-frequency stimulation, as well as a marked slowed-down recovery from depression. This excitatory/inhibitory balance in the temporal domain of short-term synaptic plasticity produced a marked hyperexcitability and enhanced network bursting behavior at network level as demonstrated by multi-electrode array recordings, also underlining the key role of short-term plasticity at excitatory and inhibitory synapses in the regulation of network excitability (71). Two further missense mutations in Syn I (A550T and T567A) located in domain D did not significantly affected phosphorylation, molecular interactions of Syn I, or neuronal development. However, the mutants were not correctly targeted to nerve terminals and, in addition, the dynamics and sizes of the readily releasable and recycling pools of SVs at synaptic terminals were impaired (67).

The Syn II mutants had distinct physiological effects. The A94fs199X-Syn II mutant was not expressed in neurons, probably because fast degradation of the aberrant protein. Both missense mutants Y236S- and G464R-Syn II were correctly expressed in Syn II KO neurons and targeted to nerve terminals. However, both mutants impaired the size of the recycling pool of SVs, leaving the readily releasable pool unaffected. Moreover, the G464R-Syn II mutant also caused an impaired axonal growth and dendritic development of neurons (68). Similar defects in neuronal development and dendritic arborization were found in neurons silenced for the ASD-associated gene CNTNAP2, with a resulting impairment of neural circuit assembly and changes in network activity, possible causes of ASD pathogenesis (72).

Synapsin KO neurons expressing the genetic variants of Syns share common defects in SV pool dynamics. Syn I and Syn II are known to control the density of SVs at the nerve terminal and regulate their availability for release differentially, with Syn I affecting both the readily releasable and recycling pools of SVs, and Syn II only affecting the latter pool. The analysis of the dynamics of exo-endocytosis in neurons expressing genetic variants of Syns reflects these distinct effects. ASD manifestations begin in the second/third year of life, a period of intense refinement, remodeling, and experience-dependent plasticity of synapses. This periods overlaps with developmental expression pattern of Syns. Impairments in SV pool dynamics, associated with defects in short-term plasticity and/or neuronal development, may thereby destabilize the key processes of assembly of neuronal networks and the balance between excitation and inhibition.

## Concluding Remarks

Synapsins are not essential for synaptic transmission, but play a key role in synaptic homeostasis and plasticity with direct consequences in network activity and excitatory/inhibitory balance. Based on the findings in human and mice, Syn genes may represent a common genetic basis for epilepsy and ASD and, accordingly, Syn KO mice can be considered a potentially interesting animal model for ASD. Similar to what occurs in children with ASDs and epilepsy, ASD-related behaviors in Syn I KO and Syn II KO mice precede the onset of seizures and epilepsy does not significantly affect the expression of the behavioral alterations in adult mice. Moreover, the non-epileptic Syn III KO mice also display some traits of social deficits. These observations lead to the idea that epilepsy and ASD follow distinct and independent pathogenic pathways, although the genetic basis appears to be largely shared by the two diseases. Although the mutations in the Syn genes found thus far account only for a limited number of ASD cases, they map into a “synaptic autism pathway” in which dysfunctions of any of the genes essential for the regulation of synapse formation, excitation/inhibition balance and activity-dependent plasticity can result in a similar ASD phenotype. Despite the inherent redundancy and robustness of mammalian biological systems, a focused dysfunction in one synaptic gene can induce secondary changes in the synaptic machinery impacting on synaptic plasticity, leading to complex dysfunctions at the circuit level associated with the appearance of the pathological phenotype. An example of this potential derangement of a complex machinery by genetic dysfunction of a single component is provided by the numerous and diverse genes implicated in phototransduction whose mutation converges toward the common clinical phenotype of *Retinitis pigmentosa* (73). In addition, it has been proposed that gene alterations and secondary dysfunctions may accumulate non-linearly in complex gene networks implicated in neural computation and higher brain functions, such as those constituting the synaptome (74, 75). In conclusion, although our map of ASD vulnerability genes is rapidly progressing, many challenges remain for the future, particularly concerning the interactions between genetic, epigenetic, and environmental factors to produce the complex ASD clinical manifestations.

## Conflict of Interest Statement

The authors declare that the research was conducted in the absence of any commercial or financial relationships that could be construed as a potential conflict of interest.
